# Anti-Cancer Effects of Carnosine—A Dipeptide Molecule

**DOI:** 10.3390/molecules26061644

**Published:** 2021-03-16

**Authors:** Monica D. Prakash, Sarah Fraser, Jennifer C. Boer, Magdalena Plebanski, Barbora de Courten, Vasso Apostolopoulos

**Affiliations:** 1 Institute for Health and Sport, Victoria University, Melbourne, VIC 3030, Australia; monica.prakash@rmit.edu.au (M.D.P.); Sarah.Fraser@vu.edu.au (S.F.); 2 Translational Immunology and Nanotechnology Research Program, School of Health and Biomedical Science, RMIT University, Melbourne, VIC 3083, Australia; jennifer.boer@rmit.edu.au (J.C.B.); magdalena.plebanski@rmit.edu.au (M.P.); 3 Department of Medicine, School of Clinical Sciences, Monash University, Clayton, VIC 3168, Australia

**Keywords:** carnosine, anti-cancer, cytokine, β-alanyl-l-histidine, immunomodulation

## Abstract

Background: Carnosine is a dipeptide molecule (β-alanyl-l-histidine) with anti-inflammatory, antioxidant, anti-glycation, and chelating properties. It is used in exercise physiology as a food supplement to increase performance; however, in vitro evidence suggests that carnosine may exhibit anti-cancer properties. Methods: In this study, we investigated the effect of carnosine on breast, ovarian, colon, and leukemic cancer cell proliferation. We further examined U937 promonocytic, human myeloid leukemia cell phenotype, gene expression, and cytokine secretion to determine if these are linked to carnosine’s anti-proliferative properties. Results: Carnosine (1) inhibits breast, ovarian, colon, and leukemic cancer cell proliferation; (2) upregulates expression of pro-inflammatory molecules; (3) modulates cytokine secretion; and (4) alters U937 differentiation and phenotype. Conclusion: These effects may have implications for a role for carnosine in anti-cancer therapy.

## 1. Introduction

U937 is a promonocytic, human myeloid leukemia cell line that was originally isolated from a histiocytic lymphoma patient [1]. U937 cells are promonocytes that exhibit many characteristics of normal monocytes and so are commonly used as a model for peripheral blood mononuclear cells (PBMCs). Though the expression levels are variable, there is little overall difference in the pattern of cluster of differentiation (CD) marker expression between PBMC and U937 cells [2]. U937 cells can also be differentiated in vitro using vitamin D_3_ or phorbol-12-myristate-13-acetate (PMA) to macrophages and dendritic cells [3].

Carnosine is a naturally occurring dipeptide molecule (β-alanyl-l-histidine) with anti-inflammatory, antioxidant, anti-glycation, and chelating properties [4]. It is found at endogenous concentrations of up to 20 mM in humans, predominantly in the brain and skeletal muscle [5]. Traditionally used in exercise physiology to increase performance, it is available over the counter as a food supplement. Animal studies suggest therapeutic benefits for many chronic diseases (e.g., type 2 diabetes, cardiovascular disease, stroke, Alzheimer’s disease, and Parkinson’s disease), but little in vivo evidence exists in humans [6].

There is, however, some in vitro evidence that suggests carnosine may exhibit anti-cancer properties. Carnosine can selectively inhibit proliferation of transformed cells [7]. Paradoxically, McFarland and Holliday had earlier reported that carnosine increased the Hayflick limit and slowed the growth of cultured human fibroblasts [8]. However, this may be explained by the metabolic differences between these two cell types; whereas most tumor cells rely heavily on glycolysis, in fibroblasts ATP synthesis predominantly occurs in the mitochondria [9]. In glioblastoma, carnosine inhibits proliferation of primary cell cultures derived from surgically removed tumors [10]. Carnosine also inhibits proliferation of gastric, colon, and ovarian cancer cell lines [11,12,13]. In this study, we sought to replicate these observations and also included breast cancer and promonocytic leukemia cells. We further determined promonocytic (U937) cell phenotype, gene expression, and cytokine secretion to determine whether these are linked to its anti-cancer properties and to identify potential pathways via which carnosine exerts its effects. In addition, U937 cells were differentiated to become monocyte/macrophage cells, and cell changes to cell surface expression were determined using flow cytometry.

## 2. Results

### 2.1. Carnosine Inhibits Cancer Cell Proliferation

**Figure 1:**
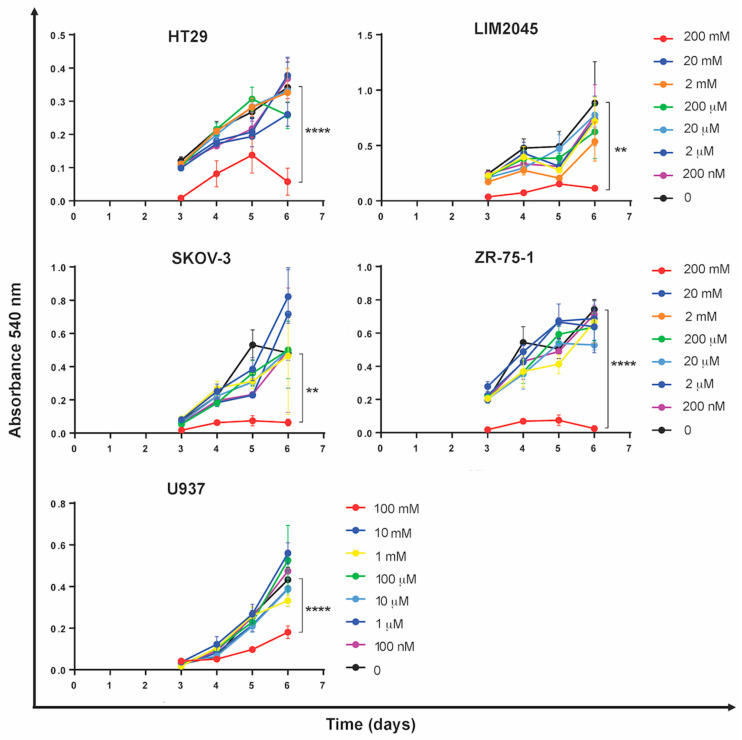
Carnosine inhibits proliferation of breast, ovarian, colon, and leukemic cancer cells. Cell proliferation was measured by 3-(4,5-dimethylthiazol-2-yl)-2,5-diphenyltetrazolium bromide (MTT) assay in the presence and absence of carnosine over 6 days. Proliferation was significantly inhibited by ≥100 mM carnosine after 6 days. Figure shows representative data from two experiments where error bars indicate standard error of the mean between triplicate wells (** *p* < 0.01, **** *p* < 0.0001).

HT29 (colon), LIM2045 (colon), SKOV-3 (ovarian), and ZR-75-1 (breast) cancer cell lines were cultured with increasing concentrations of carnosine from 0 to 200 mM for 6 days. U937 cells were cultured with a carnosine dose range from 0 to 100 mM for 6 days. At 100–200 mM carnosine for each line, proliferation was notably inhibited by day 5, which was even more pronounced at day 6 (Figure 1). At 10–20 mM carnosine, there was no significant difference in proliferation between treated and untreated cells. However, in U937 cells, the proliferation curves show some evidence of titration. Therefore, this cell line was selected for further analysis.

### 2.2. Carnosine Upregulates Expression of Proinflammatory Molecules in U937 Cell

**Figure 2:**
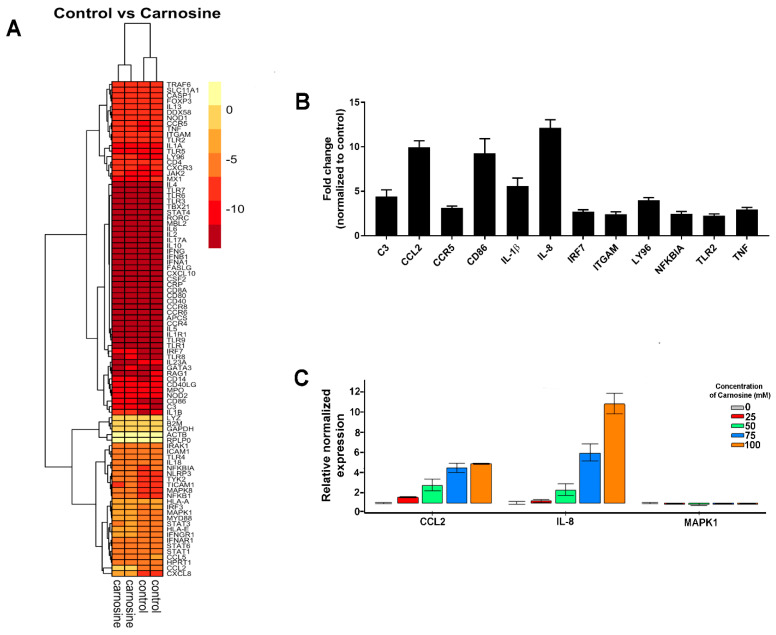
Carnosine increases gene expression of anti-cancer molecules in U937 cells. U937 cells were cultured in the presence (100 mM) or absence (control) of carnosine for 24 h, and gene expression was analyzed using the RT^2^ Profiler™ PCR Array of Human Innate Adaptive Immune Responses kit. (**A**) Differential gene expression was observed between control and carnosine groups. (**B**) Carnosine upregulated gene expression of C3, CCL2, CCR5, CD86, IL-1β, IL-8, ITGAM, LY96, NFKBIA, TLR2, and TNF in U937 cells. Figure shows mean ± standard error of the mean from 2 independent experiments. (**C**) The differential expression of IL-8, CCL2, and MAPK1 was confirmed by RT-qPCR over a range of carnosine concentrations (0–100 mM).

Gene array data showed differential regulation of a number of genes following exposure of U937 cells to carnosine (Figure 2A). Significant upregulation in gene expression was observed for IL-8, CCL2, CD86, IL-1β, CCR5, Ly96, IRF7, C3, and TNF (Figure 2B). IL-8, CCL2, and CCR5 are involved in chemotaxis; similarly, C3 is important for monocyte adhesion. CD86, IL-1β, Ly96, and TNFα are all involved in inflammatory pathways. We note that gene expression events that would result in changes in proliferation/protein expression would occur much earlier, and therefore, gene expression was assessed at an earlier time point, i.e., 24 h rather than 6 days.

The results of the array were confirmed by RT-qPCR of CCL2, IL-8, and MAPK1 (Figure 2C). These genes were specifically selected for the validation study because CCL2 and IL-8 showed the largest fold-changes in the array, and MAPK1 by comparison exhibited no change. The RT-qPCR showed that CCL2 and IL-8 gene upregulation was observed at 25 mM carnosine, and that the effect became more pronounced with increasing concentrations in a dose-dependent manner.

### 2.3. Carnosine Modulates Cytokine Secretion from U937 Cells

**Figure 3:**
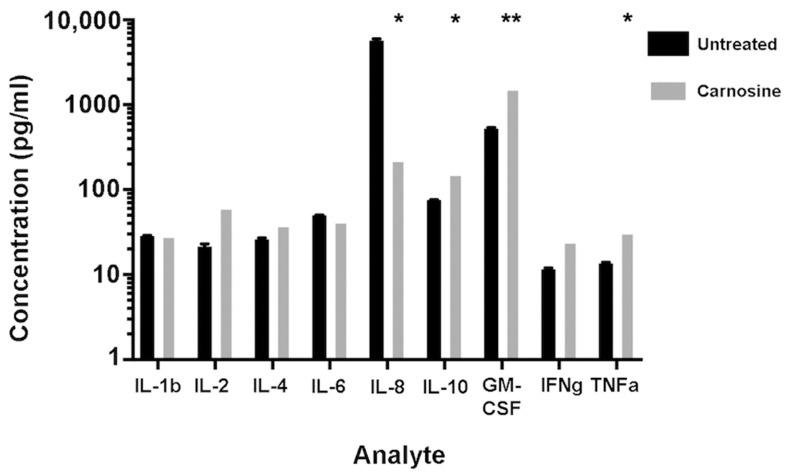
Carnosine increases secretion of IL-10, GM-CSF, and TNFα and decreases secretion of IL-8. U937 cells were cultured in the presence or absence of 100 mM carnosine for 5 days (120 h). Culture supernatant was collected, and cytokine secretion was assessed via Bioplex assay. Carnosine increased secretion of IL-10, GM-CSF, and TNFα and decreased IL-8 secretion in U937 cells. Figure shows representative data where error bars indicate standard error of the mean between replicate wells (* *p* < 0.01, ** *p* < 0.001).

U937 cells were cultured in the presence or absence of 100 mM carnosine for 5 days, and culture supernatants were analyzed for the presence of cytokines. Once the raw data were adjusted to reflect the cell number in culture, it was found that carnosine increased the secretion of IL-10, GM-CSF, and TNF-α (Figure 3). IL-10 is well-established as an anti-inflammatory cytokine. GM-CSF is primarily implicated in innate cell expansion, but also primes mature immune cells, such as neutrophils. TNFα is involved in acute inflammation and has a critical autocrine role for monocytes. Interestingly, carnosine also decreased IL-8 secretion (Figure 3). IL-8 is important in mediating adhesion of monocytes to the vascular endothelium, allowing recruitment to sites of inflammation.

### 2.4. Carnosine Alters U937 Differentiation and Phenotype

**Figure 4:**
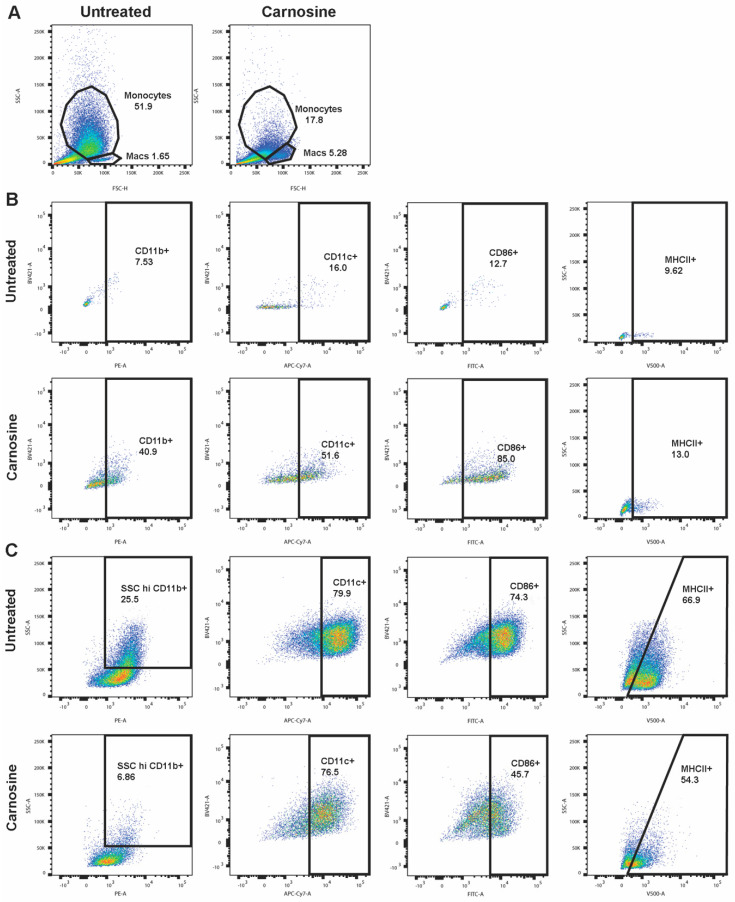
Carnosine inhibits U937 differentiation to monocytes and alters the cellular phenotype. U937 cells were differentiated by exposure to VitD_3_ for 72 h and then cultured in 100 mM carnosine for a subsequent 5 days (120 h). Cells were labelled using CD14-BV421, CD11b-PE, CD11c-APC/Cy7, CD86-AlexaFluor488, and MHCII-BV510 and analyzed by flow cytometry. Two distinct populations were identified based on forward scatter (FSC) and side scatter (SSC), identified as monocyte-like (Monocytes) and macrophage-like (Macs) (**A**). Carnosine affected each population differently, altering the expression profile in both macrophage-like (**B**) and monocyte-like (**C**) cells. The figure shows representative data from one experiment.

U937 can be differentiated to monocytes using VitD_3_. Upon culture for 72 h with VitD_3_, U937 exhibit increased side scatter (SSC), indicative of a monocyte-like phenotype. However, in the presence of carnosine, this population is significantly smaller, with an increase in a second population with larger cell size, but lower SSC (Figure 4A). Upon further characterization, this carnosine-induced population is CD11b^+^CD11c^+^CD86^+^ MHCII^+^, indicating a macrophage-like phenotype (Figure 4B).

In the monocyte-like population (Figure 4C), carnosine decreases CD11b, CD86, and MHCII expression, whereas in the macrophage-like population (Figure 4B), CD11b, CD11c, CD86, and MHCII are increased. This may suggest that carnosine is promoting differentiation to macrophages, rather than to monocytes.

## 3. Discussion

Herein, we present observations on the effect of carnosine on a number of cancer cell lines, with further examination of U937 monocyte-like cells. Carnosine (1) inhibits breast (ZR-75-1), ovarian (SKOV-3), colon (HT29, LIM2045), and leukemic (U937) cancer cell proliferation; (2) upregulates expression of pro-inflammatory molecules; (3) modulates cytokine secretion; and (4) alters U937 differentiation and phenotype. Similar to its effect on other cancer cell lines, carnosine also exerts its anti-cancer effect on U937 by inhibiting proliferation at 100 mM. Importantly, upon visual observation of cell cultures, there was no evidence of cellular stress or cell death (i.e., blebbing or cell debris). Although the carnosine concentrations used may seem high, carnosine is found endogenously in human tissue at concentrations of up to 20–30 mM [14,15] and has been used in human clinical trials to treat a range of conditions (e.g., neurodegenerative disease, type 2 diabetes, cardiovascular disease, stroke) at doses of up to 1500–2000 mg/day [16,17,18]. Moreover, although carnosine exerted its effect on a several different cell lines, the concentration at which growth inhibition was observed varied, demonstrating sensitivity in a cell-specific manner. This may be explained by metabolic differences between the different cell types; more specifically, whether they rely more heavily on glycolysis or mitochondrial ATP synthesis for energy [9].

U937 cells are a myeloid leukemia, so have the capacity to secrete many cytokines and chemokines either constitutively or in response to specific stimuli. The genes upregulated in response to carnosine are largely inflammatory mediators, so may contribute to its anti-cancer properties by making U937 more detectable to the immune system. In addition, IL-1β is a pro-inflammatory cytokine that has previously been shown to have tumoricidal activity and repress tumor growth [19]; thus, its increased gene expression may contribute to the anti-proliferative effects of carnosine. The gene expression of the chemoattractant CCL2 was also increased, and is known to exert both pro- and anti-tumor effects [20]. CCR5 gene expression was also increased, and CCR5 is commonly known to induce cancer growth and many anti-cancer clinical studies aim to block CCR5 expression on cancer cells [21]; however, there is considerable controversy regarding its role in cancer progression. A number of studies have shown either pro- or anti-cancer effects of CCR5, and this discrepancy may be a result of the type of cancer cells and the context in which cancer cells originate [22,23]. Here, we show that upregulation of CCR5 in the presence of carnosine might be involved in anti-tumor effects, concurrent with existing data. In addition, complement C3 gene expression was also increased in the presence of carnosine, and although C3 is generally known to promote cancer cell growth, there are studies showing a dual role of complement in cancer and its anti-cancer effects [24]. Furthermore, interferon regulatory factor 7 (IRF7) gene expression was upregulated in the presence of carnosine; IRF7 is known to decrease cancer growth and metastasis. In fact, silencing IRF7 in breast cancer cell lines enhances growth and restoring IRF7 expression reduces metastasis [25]; similarly, in prostate cancer in mice, overexpression of IRF7 significantly reduces metastasis [26].

The cytokine secretion assay revealed that carnosine increased secretion of IL-10, GM-CSF, and TNFα and decreased secretion of IL-8. It has been reported that high levels of IL-10 in tumors inhibit tumor metastasis [27]; tumor cell lines transfected with IL-10 show inhibition of cell growth by increasing IFNγ from CD8+ T cells [28], and IL-10 transgenic mice stimulate CD8+ T cells and limit the growth of immunogenic tumor cells in vivo [29]. Thus, although IL-10 is known as an anti-inflammatory, immunosuppressive cytokine, it has also been shown to have immunostimulatory activities that inhibit tumor cell growth. In fact, recombinant PEGylated IL-10 inhibits tumor cell growth in mice [30]. Therefore, increased levels of IL-10 by U937 cells in the presence of carnosine may suggest an anti-cancer mechanism. Furthermore, GM-CSF secretion by tumor cells has been shown to boost anti-tumor immunity, and a number of human clinical studies are using recombinant GM-CSF injections into the tumor, GM-CSF fused with tumor-associated proteins in cancer vaccines, and anti-cancer DNA vaccines incorporating GM-CSF [31]. GM-CSF regulates cancer cell growth, leading to immunosuppression in the tumor microenvironment. Thus, the increased secretion of GM-CSF in the presence of carnosine contributes to its anti-proliferative potential. In addition, carnosine increases the secretion and gene expression of pro-inflammatory cytokine TNFα, a known anti-cancer agent with the capacity to induce cancer cell death [32]. Although IL-8 is predominantly known for its immune (neutrophil) chemo-attractive properties, it has also been reported to play a role in tumor progression and metastasis in a number of human cancers by regulating angiogenesis and cytokine secretion from tumor infiltrating macrophages in the tumor microenvironment [33]. The decreased secretion of IL-8 noted by U937 cells in the presence of carnosine is potentially inhibitory to cancer progression. It is clear that the combined increased gene expression of IL-1β, the secretion of IL-10, GM-CSF, and TNFα, and decreased secretion of IL-8 contribute to the anti-proliferative effects of carnosine on U937 promonocytic leukemia cells.

In addition, carnosine is a known anti-glycating molecule with a number of antioxidant properties and the capacity to act as a ROS scavenger [4]. These actions can potentially counteract the oxidative stress and resultant chronic inflammation that have become known as hallmarks of cancer [34] and so may contribute to the anti-cancer effect observed in this study. This is an area worth exploring further.

Interestingly, the gene expression data presented in Figure 2 show that IL-8 gene expression was upregulated by carnosine, whereas in Figure 3, IL-8 secretion was decreased. However, gene expression was measured at a much earlier time point (24 h) than cytokine secretion (5 days), which may account for these seemingly disparate observations. Furthermore, it is possible that the IL-8 protein was expressed but not secreted and stored intracellularly instead, potentially requiring a second stimulus for secretion. This would also account for any discrepancy.

Finally, the altered U937 phenotype and differentiation observed in Figure 4 may also contribute to the anti-cancer effects of carnosine. U937 cells are a promonocytic leukemia and so are characteristically undifferentiated. In carnosine-treated cells, differentiation and phenotype are altered, increasing expression of CD11b, CD11c, CD86, and MHCII, thus enabling the promonocytic leukemia cells to be more visible to the immune system.

## 4. Materials and Methods

### 4.1. Cell Culture

HT29 (colon), LIM2045 (colon), SKOV-3 (ovarian), U937 (promonocytic leukemia), and ZR-75-1 (breast) cancer cell lines were maintained at sub-confluence in complete RPMI (Roswell Park Memorial Institute-1640 media supplemented with 10% fetal bovine serum, penicillin/streptomycin and l-glutamine; all purchased from Sigma-Aldrich, St Louis, MO, USA) and 5% CO_2_ at 37 °C.

### 4.2. Proliferation Assay

Cell lines were cultured in complete RPMI and varying concentrations of carnosine from 0 to 200 mM dissolved directly in the culture media in quadruplicate plates for up to 6 days. Culture media were replenished at day 3 to maintain nutrient supply; the same method was applied to all cell lines. On days 3–6, MTT (3-(4,5-dimethylthiazol-2-yl)-2,5-diphenyltetrazolium bromide) (Sigma-Aldrich) assay was performed on one plate each day to quantitate proliferation [35]. Briefly, all except 50 μL media was carefully removed from each well. An additional 50 μL of 5 mM MTT in phosphate buffered saline (PBS) was added to each well, and cells were resuspended by pipetting and incubated in MTT at 37 °C for 4 h (h). Dimethyl sulfoxide (Sigma-Aldrich, Melbourne VIC Australia) (100 μL) was added to each well for 10 min at 37 °C. Each well was pipetted before absorbance was read at 540 nm using a spectrophotometer (Bio-Rad microplate reader 6.0).

### 4.3. Gene Array

U937 cells were cultured in the presence or absence of 100 mM carnosine for 24 h and RNA was extracted using TRIzol (Invitrogen, Thermo Fisher Scientific, VIC Australia) followed by purification using RNeasy Mini Kit (Qiagen, Germany), including on-column DNase-treatment. RNA was quantified using Qubit™ RNA BR Assay Kit and RNA integrity number (RIN) established using an Agilent 2100 Bioanalyzer (all RIN were ≥9.5). Samples were processed according to manufacturer’s instructions using the RT^2^ Profiler™ PCR Array Human Innate and Adaptive Immune Responses (Qiagen). Expression levels were normalized to the mean of the reference genes present on the array (*GAPDH, ACTB, B2M, HPRT1,* and *RPLP0*) and fold change was calculated using the 2^ (-delta delta C_T_) formula via the Qiagen GeneGlobe web portal.

The pheatmap (version 1.0.12) data package for R software (version 4.0.2) was used together with RColorBrewer (version 1.1–2) for heatmap analysis. Plots were generated from log2 transformed DeltaCt values from 2 PCR arrays. The cluster function was applied to indicate genes that have expression similarity.

The differential expression of three genes—namely, interleukin 8 (IL-8/CXCL8) (NM_000584), C-C motif chemokine ligand 2 (CCL-2) (NM_002982), and mitogen-activated protein kinase 1 (MAPK1) (NM_138957)—was confirmed by RT-qPCR over a range of carnosine concentrations (0–100 mM). cDNA was reverse transcribed from DNase-treated RNA (1 µg) using SuperScript IV VILO Master Mix (Life Technologies, ThermoFisher). PCR was performed using SsoAdvanced™ SYBR Green Supermix (Bio-rad) using primers from Integrated DNA Technologies (IDT): CCL-2 forward 5′-AGC AGC CAC CTT CAT TCC-3′, reverse 5′-GCC TCT GCA CTG AGA TCT TC-3′; CXCL8 forward 5′-GAG ACA GCA GAG CAC ACA AG-3′, reverse 5′-CTT CAC ACA GAG CTG CAG AA-3′ and MAPK1 forward 5′-CAT TCA GCT AAC GTT CTG CAC-3′, reverse 5′-GTG ATC ATG GTC TGG ATC TGC-3′. PCR analysis was performed in duplicate. Normalized relative expression (fold change) was determined relative to control (0 mM carnosine) and normalized to two stably expressed reference genes (actin beta, ACTB and glucuronidase beta, GusB) using ΔΔCT data analysis function in CFX™ Maestro (Bio-rad), version 1.1.

### 4.4. Cytokine Secretion

U937 cells were cultured at an initial density of 5 × 10^3^ cells/mL, with or without 100 mM carnosine for 5 days. Supernatants were collected, centrifuged, and frozen at −80 °C until assayed. Cytokine secretion was assessed via Bioplex assay (Bio-Rad, USA) according to manufacturer’s instructions.

### 4.5. Flow Cytometry

U937 cells were differentiated to monocytes/macrophages by exposure to 1,25-dihydroxyvitamin D3 (VitD_3_) for 72 h and then cultured in 100 mM carnosine for a subsequent 5 days (120 h). Cells were then harvested and labelled using CD14-BV421, CD11b-PE, CD11c-APC/Cy7, CD86-AlexaFluor488, MHCII-BV510 and analyzed using a BD FACSCanto flow cytometer. Data analysis was performed using FlowJo software (Treestar, USA).

### 4.6. Statistical Analysis

The statistical analyses for the cancer cell line proliferation assays and cytokine secretion by 9-plex human bioplex assay were performed using GraphPad Prism software, version 7.0e. The student *t*-test was performed using the Holm–Sidak method with alpha = 0.05, where *p* < 0.05 was regarded as statistically significant. Data are presented as mean ± standard error of the mean (SEM). To analyze the human innate and adaptive immune response genes, RT^2^ Profiler PCR array (Qiagen software) was used based on normalization with reference genes.

## 5. Conclusions

In this study, we observed that carnosine significantly inhibits proliferation of breast, ovarian, colon, and leukemic cancer cell lines. Further studies revealed additional effects regarding U937 cell phenotype, gene expression, and cytokine secretion. Together, the observation of increased secretion of IL-10, GM-CSF, and TNFα, decreased secretion of IL-8, increased gene expression of IL-8, CCL2, CD86, IL-1β, CCR5, Ly96, IRF7, C3, and TNFα, and increased expression of cell surface markers CD11b, CD11c, CD86, and MHCII all have implications for the anti-proliferative properties of carnosine, which should be further explored in anti-cancer therapy.

## Data Availability

The data is available upon request.
